# Somatostatin Receptor Imaging with [^18^F]FET-βAG-TOCA PET/CT and [^68^Ga]Ga-DOTA-Peptide PET/CT in Patients with Neuroendocrine Tumors: A Prospective, Phase 2 Comparative Study

**DOI:** 10.2967/jnumed.123.266601

**Published:** 2024-03

**Authors:** Suraiya Dubash, Tara D. Barwick, Kasia Kozlowski, Andrea G. Rockall, Sairah Khan, Sameer Khan, Siraj Yusuf, Angela Lamarca, Juan W. Valle, Richard A. Hubner, Mairéad G. McNamara, Andrea Frilling, Tricia Tan, Florian Wernig, Jeannie Todd, Karim Meeran, Bhavesh Pratap, Saleem Azeem, Michael Huiban, Nicholas Keat, Jingky P. Lozano-Kuehne, Eric O. Aboagye, Rohini Sharma

**Affiliations:** 1 Department of Surgery and Cancer, Imperial College London, London, United Kingdom;; 2 Department of Imaging, Imperial College Healthcare NHS Trust, London, United Kingdom;; 3 Radiology and Nuclear Medicine Department, Royal Marsden NHS Foundation Trust, London, United Kingdom;; 4 Division of Cancer Sciences, University of Manchester, Manchester, United Kingdom;; 5 Department of Medical Oncology, The Christie NHS Foundation Trust, Manchester, United Kingdom;; 6 Department of Endocrinology, Imperial College Healthcare NHS Trust, London, United Kingdom;; 7 Invicro-London, Imperial College London, London, United Kingdom; and; 8 Population Health Sciences Institute, Faculty of Medical Sciences, University of Newcastle, Newcastle, United Kingdom

**Keywords:** [^18^F]FET-βAG-TOCA, [^68^Ga]Ga-DOTA-peptide, PET, neuroendocrine tumors, somatostatin receptor

## Abstract

There is a clinical need for ^18^F-labeled somatostatin analogs for the imaging of neuroendocrine tumors (NET), given the limitations of using [^68^Ga]Ga-DOTA-peptides, particularly with regard to widespread accessibility. We have shown that [^18^F]fluoroethyl-triazole-[Tyr^3^]-octreotate ([^18^F]FET-βAG-TOCA) has favorable dosimetry and biodistribution. As a step toward clinical implementation, we conducted a prospective, noninferiority study of [^18^F]FET-βAG-TOCA PET/CT compared with [^68^Ga]Ga-DOTA- peptide PET/CT in patients with NET. **Methods:** Forty-five patients with histologically confirmed NET, grades 1 and 2, underwent PET/CT imaging with both [^18^F]FET-βAG-TOCA and [^68^Ga]Ga-peptide performed within a 6-mo window (median, 77 d; range, 6–180 d). Whole-body PET/CT was conducted 50 min after injection of 165 MBq of [^18^F]FET-βAG-TOCA. Tracer uptake was evaluated by comparing SUV_max_ and tumor-to-background ratios at both lesion and regional levels by 2 unblinded, experienced readers. A randomized, blinded reading of both scans was also then undertaken by 3 experienced readers, and consensus was assessed at a regional level. The ability of both tracers to visualize liver metastases was also assessed. **Results:** A total of 285 lesions were detected on both imaging modalities. An additional 13 tumor deposits were seen in 8 patients on [^18^F]FET-βAG-TOCA PET/CT, and [^68^Ga]Ga-DOTA-peptide PET/CT detected an additional 7 lesions in 5 patients. Excellent correlation in SUV_max_ was observed between both tracers (*r* = 0.91; *P* < 0.001). No difference was observed between median SUV_max_ across regions, except in the liver, where the median tumor-to-background ratio of [^18^F]FET-βAG-TOCA was significantly lower than that of [^68^Ga]Ga-DOTA-peptide (2.5 ± 1.9 vs. 3.5 ± 2.3; *P* < 0.001). **Conclusion:** [^18^F]FET-βAG-TOCA was not inferior to [^68^Ga]Ga-DOTA-peptide in visualizing NET and may be considered in routine clinical practice given the longer half-life and availability of the cyclotron-produced fluorine radioisotope.

## Visual Abstract

**Figure:**
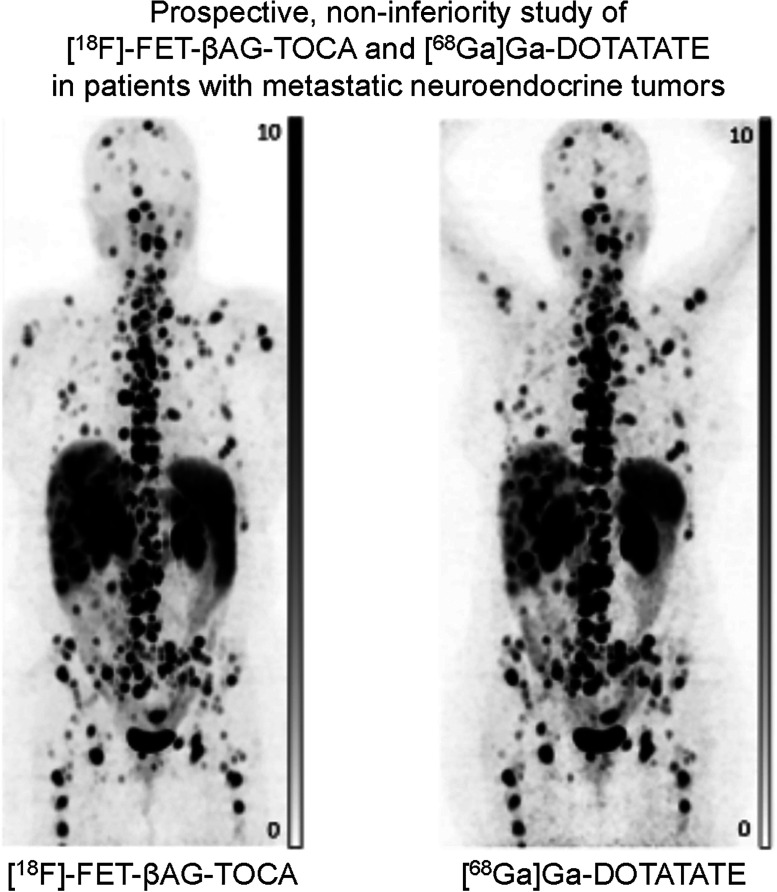

Neuroendocrine neoplasms (NEN) are a heterogeneous group of malignancies arising from cells of the diffuse neuroendocrine system. Accurate diagnosis of primary lesion and staging the extent of disease dictates both management and prognosis, whereby patients with limited disease can undergo radical locoregional therapy, including surgery or ablation with curative intent, whereas systemic therapy is reserved for those with metastatic disease given with palliative intent (1). Accurate imaging is crucial. As 20%–50% of patients with NEN will have metastatic disease at presentation (2), there is a need for an imaging methodology that is both sensitive and widely accessible.

A unique characteristic of NEN is the expression of somatostatin receptors (SSTRs) on the tumor surface (3). The presence of SSTRs has long been exploited for imaging NEN initially with planar or SPECT imaging using [^111^In]In-diethylenetriaminepentaacetic acid-octreotide and, more recently, with PET/CT using radiolabeled somatostatin analogs (SSAs). PET imaging has greater sensitivity, enhanced resolution, and better accuracy in detecting NEN compared with SPECT imaging (4,5). The most commonly used PET tracers used for the visualization of NEN are SSAs labeled with [^68^Ga]Ga-DOTA-peptides, including [^68^Ga]Ga-DOTA-0-Tyr3-octreotate ([^68^Ga]Ga-DOTATATE) and [^68^Ga]Ga-DOTA-0-Phe1-Tyr3-octreotide ([^68^Ga]Ga-DOTATOC). Defining the presence of SSTRs on the tumor surface is also important for therapeutic decision making, whereby patients with SSTR-positive NEN on [^68^Ga]Ga-DOTA PET may be candidates for [^177^Lu]Lu-DOTA0-Tyr3-octreotate ([^177^Lu]Lu-DOTATATE), a targeted radiotherapeutic that significantly improves progression-free survival in patients with metastatic disease (6).

Although [^68^Ga]Ga-DOTA analogs have good resolution, the availability and scalability of production is limited due to the necessity of an on-site generator pertaining to the short half-life of [^68^Ga]Ga. Furthermore, the [^68^Ga]Ga-radiometal may accumulate within the uncinate process of the pancreas, leading to a false-positive diagnosis (7). Clinically, a [^18^F]F-radioligand would overcome the limited capacity of [^68^Ga]Ga-DOTA production while exploiting existing worldwide cyclotron manufacturing. We developed a novel [^18^F]F-octreotate radioligand, [^18^F]fluoroethyl-triazole-[Tyr^3^]-octreotate ([^18^F]FET-βAG-TOCA) (8), to obviate these limitations of [^68^Ga]Ga-DOTA ligands. Previously, we showed that tumor uptake of [^18^F]FET-βAG-TOCA was superior to that of [^68^Ga]Ga-DOTATATE in vivo with good spatial resolution (9). Clinically, [^18^F]FET-βAG-TOCA has favorable dosimetry and biodistribution (8). We therefore performed a prospective study, the primary objective of which was to assess uptake of [^18^F]FET-βAG-TOCA both at lesion and regional levels. Evaluation of interreader agreement between [^18^F]FET-βAG-TOCA and [^68^Ga]Ga-DOTA-peptide PET was assessed as a secondary endpoint.

## MATERIALS AND METHODS

### Study Design and Participants

A prospective, multicenter, open-label, single-arm comparative imaging study consisting of an initial safety run phase (part A) followed by a noninferiority phase (part B) was conducted. The safety and biodistribution study (part A) has been reported (8). Patients from part A (*n* = 9) were included in the noninferiority analysis. Key eligibility criteria include histologically confirmed diagnosis of locally advanced or metastatic grade 1 or 2 neuroendocrine tumors (NET), measurable disease with at least 1 lesion with longest diameter ≥ 10 mm on conventional imaging, and positive SSTR imaging within 6 mo of study enrollment with [^68^Ga]Ga-DOTA-peptide PET. Patients were not required to stop SSAs before either PET scan. Patients were recruited from 2 U.K. European Neuroendocrine Tumor Society Centers of Excellence, Imperial College Health Care NHS Trust and Christie NHS Foundation Trust, Manchester. All diagnostic tissue samples underwent central pathology review to assess eligibility. The study was approved by the Leeds East, Yorkshire and Humber National Research Committee (13/YH/0281). The administration of radioactivity was approved by the Administration of Radioactive Substances Advisory Committee (United Kingdom) (RPC 630/2892/30595). The Medicines and Health Care Products Regulatory Agency (United Kingdom) gave permission to administer the investigational medicinal product (European Clinical Trials no. 2013-003152-20). All patients provided written informed consent. The study was conducted in accordance with the Declaration of Helsinki and registered with EudraCT (2013-003152-20).

### Procedures

#### PET Imaging Protocol

At Imperial College Health Care NHS Trust, clinical PET/CT imaging was performed using [^68^Ga]Ga-DOTATATE, as previously described (10) (mean dose injected, 134.1 MBq; mean uptake period, 37.7 min [range, 27–82 min]). At Christie NHS Foundation Trust, imaging was performed using [^68^Ga]Ga-DOTANOC PET (mean dose injected: 136.2 MBq and mean uptake period of 65.2 min (range, 30–82 min) and a single case [^68^Ga]Ga-DOTATOC (dose, 143 MBq; uptake time, 75 min). No clinically significant differences in DOTA tracers have been reported (11,12), and these patients were all included for the primary analysis. Imaging with [^18^F]FET-βAG-TOCA was conducted after [^68^Ga]Ga-DOTA-peptide PET in most cases.

[^18^F]FET-βAG-TOCA was synthesized by Invicro-London (8); the mean dose injected was 157.7 MBq, with a mean uptake period of 37.4 min (range, 30–51 min). Images were acquired on a Siemens Biograph 6 TruePoint PET/CT scanner (with TrueV; extended field of view) at 50 min after injection (8). An attenuation CT scan was obtained from vertex to midthigh, immediately followed by a PET emission study at 4 min per bed position (CT settings: tube potential, 130 kV; exposure, 15 effective mAs; pitch, 1.5; slice thickness, 5 mm; rotation time, 0.6 s). Images were reconstructed using the ordered-subsets expectation maximization algorithm (4 iterations and 8 subsets) with corrections for dead time, scatter, attenuation, and radioactive decay. All images were viewed on a dedicated PET workstation (Hermes Medical Solutions).

#### Image Interpretation

Images were reviewed by 2 observers: a radiation oncologist with greater than 15 y of experience in imaging and tumor outlining and an experienced radiologist (with dual accreditation in radiology and nuclear medicine) with greater than 20 y of experience. To ensure a methodical and consistent approach, comparison of [^18^F]FET-βAG-TOCA with [^68^Ga]Ga-DOTA-peptide PET/CT on a patient-by-patient and lesion-by-lesion analysis was performed. Due to the large number of metastases, lesions were analyzed within the context of anatomic regions. Seven regions were defined as being the most common sites for both primary tumors and metastases: head and neck, lung, liver, pancreas, abdomen/pelvis, bone and lymph nodes. Any organ with greater than 5 lesions were truncated at 5 target lesions as in previous studies (13,14). SUV measurements (SUV_max_, SUV_mean_, and tumor-to-background ratio [TBR]) were obtained for lesion-by-lesion analysis by manually outlining whole tumor volumes on side-by-side analysis of both studies to ensure, in cases with innumerable lesions, that the same lesions were selected for comparative quantitative analysis. For comparative SUV analysis, only those lesions that were visible on both [^18^F]FET-βAG-TOCA and [^68^Ga]Ga-DOTA-peptide PET/CT were included reference (normal background) tissue were outlined using a spheric reference volume of interest (3 cm^3^ for background liver; 2 cm^3^ for spleen, bone, and mediastinal blood pool); 1 cm^3^ for spheric volumes in the pancreas and sum of 3 slices manually drawn around each adrenal gland). TBR was calculated using tumor lesion SUV_max_/background tissue SUV_mean_ using background liver for liver metastases, background bone marrow for bone metastases, and background mediastinal blood pool for soft-tissue, nodal, and pulmonary metastases.

As the presence of liver metastases is an independent prognostic factor (15), subgroup analysis of SUV and TBR measurements of the liver lesions based on tumor size was performed.

#### Independent Reader Evaluations

PET/CT scans were reviewed by 3 independent imaging experts to obtain an objective interreader lesion detection rate between [^18^F]FET-βAG-TOCA PET/CT and [^68^Ga]Ga-DOTA-peptide PET/CT scans. To avoid recall bias, [^18^F]FET-βAG-TOCA PET/CT and [^68^Ga]Ga-DOTA-peptide PET/CT scans for each subject were reviewed at least 4 wk apart in random order. Readers were blinded to clinical details, type of scan and results of other imaging modalities. Readers documented the presence or absence of lesions in each of the 7 previously defined areas. Comparison was made between individual readers across both imaging modalities for interobserver agreement. After locking findings, readers then performed a final side-by-side visual analysis of the 2 sets of scans to document any discordant lesions detected on 1 scan and not the other, to arrive at consensus between the 3 readers.

Clinical cross-sectional imaging (contrast-enhanced CT or MRI) performed within 3 mo of [^18^F]FET-βAG-TOCA was reviewed by a single experienced observer with more than 20 y of experience.

### Statistical Analysis

A total of 56 patients were required based on a hypothesized 90% sensitivity, a noninferiority margin of 10%, power of 80%, and a level of significance of 5%. Descriptive statistics such as the median and interquartile range were calculated for numeric outcomes. Wilcoxon test was used for comparison of results. Pearson linear correlation test was used to evaluate correlation between SUV_max_ values. Groups were compared using the χ^2^ test. The Cohen κ and the Fleiss κ were used to determine the level of agreement among 2 and more than 2 readers of [^18^F]FET-βAG-TOCA and [^68^Ga]Ga-DOTA-peptide PET/CT, respectively. A *P* value lower than 0.05 was taken to be significant. All statistical analyses were performed using SPSS version 27.0 (IBM Inc.) and Stata 16 (StataCorp LLC).

## RESULTS

### Baseline Characteristics

A total of 56 patients were enrolled to the study. Eleven patients were excluded from the primary analysis: 2 patients underwent octreotide scan, 6 patients did not have a [^68^Ga]Ga-DOTA-peptide PET/CT within 6 mo of [^18^F]FET-βAG-TOCA PET/CT and in a further 3 patients, central pathology review after [^68^Ga]Ga-DOTA-peptide and [^18^F]FET-βAG-TOCA imaging reported high-grade neuroendocrine carcinoma. A total of 45 patients were included in the final analysis. Four patients had a [^68^Ga]Ga-DOTANOC PET/CT and 1 patient had [^68^Ga]Ga-DOTATOC PET/CT. All remaining patients underwent [^68^Ga]Ga-DOTATATE PET/CT. The median age of the enrolled population was 57 y (range, 29–81 y) and most had a diagnosis of small-bowel (44%) NET. All patients had either locally advanced (9%) or metastatic disease (91%), the commonest site of metastases being the liver (58%). Demographics and clinical characteristics of the study population are presented in Table 1. The median interval between [^18^F]FET-βAG-TOCA and [^68^Ga]Ga-DOTA PET/CT was 77 d (range, 6–180 d).

**TABLE 1.** Baseline Characteristics of Patient Cohort (*n* = 45)

Variable	Value*
Age (y)	
Median	57
Range	29–81
Sex	
Male	23 (51)
Female	22 (49)
Stage	
Locally advanced	4 (9)
Metastatic	41 (91)
Site of primary tumor	
Pancreas	15 (33)
Small bowel	20 (44)
Lung	3 (7)
Other	7 (16)
Grade	
1	15 (33)
2	21 (47)
Unknown	9 (20)
Site of metastatic disease	
Liver	27 (60)
Bone	12 (27)
Nodes	10 (22)
Lung	3 (7)
Other	17 (38)
Median Ki-67 (%)	3 (7)^†^
Chromogranin A (ng/mL)	72 (92)^†^
Previous treatment	
Surgery	24 (53)
Somatostatin analogs	20 (44)
Chemotherapy	9 (20)
PRRT	7 (16)
RFA	6 (13)
Other	3 (7)

* Data are reported as numbers of patients, with percentages of patients in parentheses.

† Value in parentheses is interquartile range.

PRRT = peptide receptor radiotherapy; RFA = radiofrequency ablation.

### Comparison of [^18^F]FET-βAG-TOCA and [^68^Ga]Ga-DOTA-Peptide PET

#### Lesion Analysis

**FIGURE 1.**
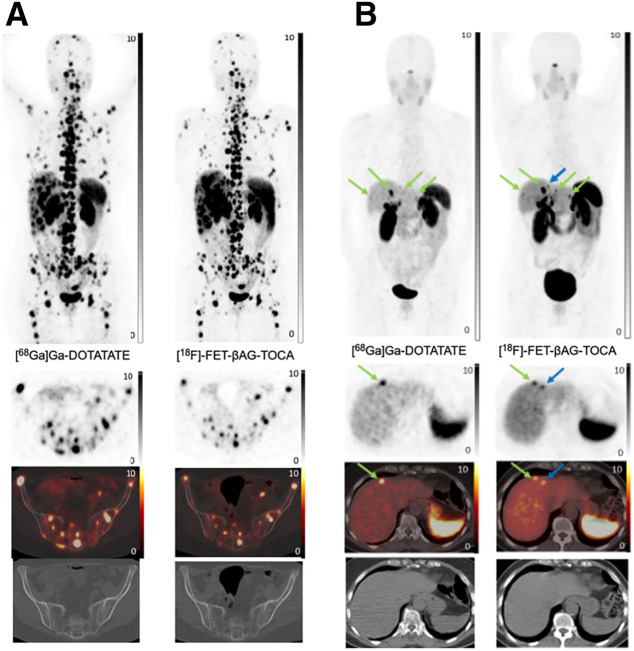
(A) Congruent imaging: [^68^Ga]Ga-DOTATATE imaging and [^18^F]FET-βAG-TOCA imaging (maximum-intensity projection [MIP], axial PET, fused and CT images) in metastatic small-bowel NEN with widespread liver and bone metastases. (B) Incongruent imaging: [^68^Ga]Ga-DOTATATE imaging and [^18^F]FET-βAG-TOCA imaging (MIP, axial PET, fused and CT images) performed 4 wk apart in metastatic ileal NEN with liver metastases (green arrows), which are more visible on [^18^F]FET-βAG-TOCA. Additional lesion is detected on [^18^F]FET-βAG-TOCA (blue arrow).

On per-lesion analysis, 285 lesions were seen both on [^18^F]FET-βAG-TOCA PET/CT and [^68^Ga]Ga-DOTA-peptide PET/CT. Most lesions were within the liver (38.6% for both imaging modalities) followed by nodal involvement (16.8%) and bone metastases (17.1%). After unblinding of readers, side-by-side visual analysis illustrated 20 discordant lesions in 11 patients; in 6 patients additional lesions were detected on [^18^F]FET-βAG-TOCA in comparison to [^68^Ga]Ga-DOTA-peptide, conversely additional lesions were detected on [^68^Ga]Ga-DOTA-peptide in 3 patients compared with [^18^F]FET-βAG-TOCA and in 2 patients there was a mixture with some lesions detected by 1 tracer and not the other and vice versa (Table 2). [^18^F]FET-βAG-TOCA detected an additional 13 lesions (6 liver metastases, 4 bone metastases, 2 nodes, and 1 small-bowel lesion) in 8 patients and [^68^Ga]DOTA-peptide PET/CT detected an additional 7 lesions (4 liver, 1 bone, 1 pancreas, and 1 node) in 5 patients (Fig. 1B).

**TABLE 2.** Discordant Lesions Between [^18^F]FET-βAG-TOCA (FETO) and [^68^Ga]Ga-DOTA-peptide (DOTA) PET/CT

Patient	Time between scans (mo)	Congruent site(s)	Lesion	Discordant lesion site	Lesion size (mm)	Scan
1	4.6	Liver	1	Liver	7	FETO
2	0.2	Liver, nodal	2	Node	5	FETO
			3	Node	5	FETO
3	4.8	Liver, peritoneal	4	Liver	7	FETO
4	0.6	Bone	5	Bone	3	FETO
5	4.3	Liver, nodal	6	Liver	10	DOTA
6	2.5	Gastric, liver	7	Liver	8	DOTA
			8	Liver	8	DOTA
7	0.2	Liver, nodal	9	Liver	12	DOTA
			10	Pancreas	13	DOTA
8	1.6	Bone, nodal	11	Bone	10	FETO
			12	Bone	4	FETO
			13	Bone	4	FETO
			14	Small bowel	5	FETO
			15	Node	15	DOTA
9	1.1	Liver	16	Liver	9	FETO
			17	Liver	9	FETO
10	3.7	Liver, nodal	18	Liver	5	FETO
11	4.4	Liver, bone	19	Bone	3	DOTA
			20	Liver	8	FETO

**FIGURE 2.**
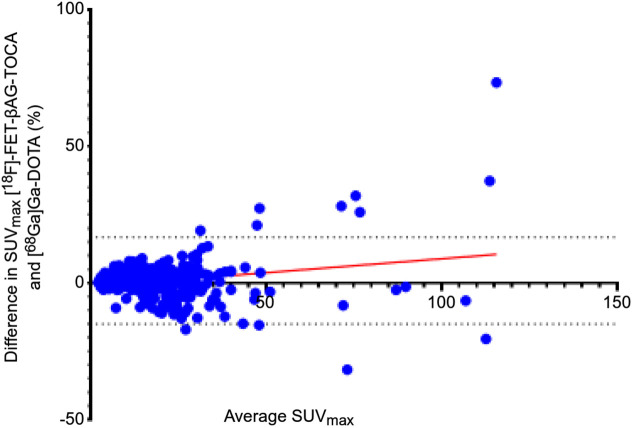
Bland–Altman plot of difference in SUV_max_ between [^18^F]FET-βAG-TOCA and [^68^Ga]Ga-DOTA-peptide.

For comparative SUV analysis, 285 lesions were included. Excellent correlation in lesion SUV_max_ between imaging modalities was observed (*r* = 0.91; *P* < 0.001) (Fig. 2). We then assessed the impact of the use of SSAs on tracer uptake. Twenty-three patients (51%) were receiving monthly injections with SSAs. No difference was observed in median SUV_max_ (± SD) of [^18^F]FET-βAG-TOCA of those receiving SSAs (19.2 ± 21.1) compared with those who were not (15.8 ± 15.9) (*P* = 0.06).

#### Regional Analysis

No significant difference was noted in the median SUV_max_ across all tumor regions between the 2 imaging modalities (Table 3). The highest median [^18^F]FET-βAG-TOCA SUV_max_ was observed in pancreatic lesions (median SUV_max_, 24.5 ± 24.9) and the lowest was observed in bone (median SUV_max_, 9.7 ± 8.8).

**TABLE 3.** Median Tumor Uptake (SUV_max_) and Tumor-to-Background Ratio (TBR) of [^18^F]FET-βAG-TOCA and [^68^Ga]Ga-DOTA-peptide per Anatomic Region

		[^18^F]FET-βAG-TOCA		[^68^Ga]Ga-DOTA-peptide			Median TBR with:		
Region	No. of lesions	Median SUV_max_	Range	Median SUV_max_	Range	*P*	[^18^F]FET- βAG-TOCA	[^68^Ga]Ga- DOTA-peptide	*P*
Head and neck	3	12.4	10.4–27.7	6.9	6.4–23.4	0.5	29.8	12.2	0.3
Liver	110	19.59	7.2–132.4	20.6	6.7–95.1	0.5	2.5	3.5	<0.001
Bone	49	9.7	2.2–37.0	7.2	1.9–38.8	0.5	12.5	10.1	0.5
Lung	11	10.4	5.3–42.1	9.4	2.2–38.0	0.9	14.6	15.4	0.4
Pancreas	28	24.5	4.2–85.8	21.9	6.4–88.4	0.8	35.2	36.6	0.6
Abdomen/pelvis	36	18.8	2.7–152.3	21.2	4.1–110.3	0.6	23.5	23.6	0.1
Lymph nodes	48	18.0	3.4–102.4	17.0	3.1–122.9	0.7	21.1	29.7	0.5

Both tracers demonstrated comparable distribution in background organs (spleen, pancreas, adrenals, bone) except for increased background hepatic activity on [^18^F]FET-βAG-TOCA PET/CT (Supplemental Fig. 1) (supplemental materials are available at http://jnm.snmjournals.org). Low physiologic uptake of [^18^F]FET-βAG-TOCA was observed, as previously described in the pituitary, salivary glands, spleen and thyroid gland (8). There was a statistical difference observed in median TBR for liver lesions with [^18^F]FET-βAG-TOCA compared with [^68^Ga]Ga-DOTA-peptide PET/CT (2.52 ± 1.88 vs. 3.50 ± . 2.35; *P* < 0.001). No other differences in regional TBR were observed (Table 3).

### Interreader Agreement

Interreader agreement across tumor sites was considered. It was possible to estimate with 95% confidence a κ-agreement of 86% with an SE of 10% assuming 90% positive ratings among raters for a total of 45 subjects. Agreement was significantly higher in the liver with [^68^Ga]Ga-DOTA-peptide (κ = 0.3) than with [^18^F]FET-βAG-TOCA (κ = 0.05) (*P* < 0.001). In particular, when considering the liver, discrepancies in reads were noted in 4 patients on [^18^F]FET-βAG-TOCA imaging, 3 of whom did not have liver metastases but were thought to be present by 1 of the 3 readers. In contrast, only 1 patient was felt to have liver metastases on [^68^Ga]Ga-DOTA-peptide PET, where none were present by 1 of the 3 readers. No significant differences in agreement were observed across other sites (Table 4).

**TABLE 4.** Interrater Agreement for [^18^F]FET-βAG-TOCA and [^68^Ga]Ga-DOTA-peptide per Anatomic Region Between All 4 Raters

	Agreement between raters 1, 2, 3, and 4 for:						
	[^18^F]FET-βAG-TOCA			[^68^Ga]Ga-DOTA-peptide			
Scan site	Agreement (%)	κ*	*P*	Agreement (%)	κ*	*P*	*P* for [^18^F]FET- βAG-TOCA vs. [^68^Ga]Ga- DOTA-peptide
Head and neck	97.4	0.2 (−0.01 to 0.3)	**<**0.001	97.8	−0.01 (−0.02 to −0.006)	0.6	0.04
Lung	97.8	−0.01 (−0.02 to −0.01)	0.6	98.9	−0.01 (NC)	0.5	NC
Liver	94.4	−0.03 (−0.05 to −0.006)	0.7	98.5	0.3 (NC)	<0.001	<0.001
Pancreas	89.3	0.2 (0.1 to 0.3)	<0.001	91.5	0.1 (−0.03 to 0.3)	0.05	0.2
Abdomen/pelvis	88.5	0.0005 (−0.07 to 0.2)	0.5	95.2	0.1 (−0.02 to 0.3)	0.04	0.3
Bone	98.5	0.3 (NC)	<0.001	100.0	NC	NC	NC
Lymph nodes	97.4	0.3 (−0.01 to 0.3)	<0.001	96.3	0.2 (−0.02 to 0.3)	0.008	0.7

* Values in parentheses are 95% CIs.

NC = noncalculable.

### Liver Metastases

As the presence of liver metastases is an independent prognostic factor, we performed subgroup analysis of uptake in the liver lesions based on lesion size (<1 cm, 1.0–2 cm, >2.1 cm). Of the 110 liver metastases, 28 lesions were smaller than 1 cm, 52 were 1–2 cm, and 30 were larger than 2.1 cm. When considering SUV_max_, no significant difference in uptake was observed with [^18^F]FET-βAG-TOCA in lesions smaller than 1 cm (15.1 ± 7.9) and those 1–2 cm (22.7 ± 19.9) compared with [^68^Ga]Ga-DOTATATE (<1 cm, 12.2 ± 6.9 [*P* = 0.2]; 1–2 cm, 22.4 ± 14.5 [*P* = 0.4]). A significantly lower median TBR was observed for lesions 1–2 cm with [^18^F]FET-βAG-TOCA (3.3 ± 2.1) compared with [^68^Ga]Ga-DOTA-peptide (4.5 ± 2.4) (*P* = 0.050. No difference was observed in median TBR for lesions smaller than 1 cm ([^18^F]FET-βAG-TOCA, 1.9 ± 0.8; [^68^Ga]Ga-DOTA-peptide, 2.3 ± 1.3) (*P* = 0.4). Overall, the [^18^F]FET-βAG-TOCA median TBR was significantly lower in the liver than the [^68^Ga]Ga-DOTA-peptide median TBR (2.5 ± 1.9 vs. 3.5 ± 2.3; *P* < 0.001).

## DISCUSSION

The superiority of [^68^Ga]Ga-DOTA-peptide PET/CT over [^111^In]In-octreotide SPECT/CT and contrast CT imaging for the visualization of NET is well established (4,5). However, the use of [^68^Ga]Ga necessitates the presence of an onsite (limited life span) generator, limiting the scalability and availability of [^68^Ga]Ga-DOTA-peptide radioligands, such that many patients are not able to access [^68^Ga]Ga-DOTA-peptide for diagnosis, treatment planning or assessment of disease progression. To alleviate these issues, we developed a GMP compliant [^18^F]F-octreotate radiopharmaceutical, [^18^F]FET-βAG-TOCA. We have previously reported [^18^F]FET-βAG-TOCA to be safe, with good dosimetry and biodistribution, that highlights tumor lesions with high contrast (8). In this prospective study, we have shown that [^18^F]FET-βAG-TOCA is excellent in detecting lesions and is not inferior to [^68^Ga]Ga-DOTA-peptide- PET/CT for the detection of NET. We have also shown the ability of [^18^F]FET-βAG-TOCA in detecting small liver lesions, an important consideration given the prognostic impact of liver metastases (15).

We observed no significant difference in tumoral SUV_max_ both on lesion and regional bases between scan types confirming the noninferiority of [^18^F]FET-βAG-TOCA for imaging NET. Observed SUV_max_ values of [^18^F]FET-βAG-TOCA are consistent with the high affinity of [^18^F]FET-βAG-TOCA for SSTR2 binding (IC_50_,1.6 ± 0.2 nM) (16). The use of SSAs had no impact on [^18^F]FET-βAG-TOCA SUV_max_, an important consideration, given the widespread use of these agents. Moreover, there was excellent correlation between the 2 tracers as confirmed by interobserver agreement across most regions.

The liver is the commonest site of metastases and is an independent prognostic factor in patients with NET (15). Background liver uptake was significantly lower with [^18^F]FET-βAG-TOCA compared with [^68^Ga]Ga-DOTA-peptide. This difference in uptake can be attributed to differences in elimination. [^18^F]FET-βAG-TOCA is eliminated by both the biliary and renal system, whereas [^68^Ga]Ga-DOTA-peptide is eliminated predominantly through the kidneys. Hepatic clearance and slow clearance through the common bile duct may contribute to the higher background uptake observed on [^18^F]FET-βAG-TOCA imaging. As a result of this difference in background uptake, a significant difference in TBR in the liver between the 2 tracers was observed. The higher liver background activity may have contributed to the difference observed on interreader agreement within the liver, whereby observers were less confident in 3 cases about the absence of metastases in “normal liver”, a concept that needs exploring in future work. However, of the 20 discordant lesions, 10 were in the liver, 6 were only detected on [^18^F]FET-βAG-TOCA and 4 with [^68^Ga]Ga-DOTA-peptide imaging. The management in these cases did not change as the patients already had multiple liver metastases.

Since ^18^F has a shorter positron range and higher positron yield than ^68^Ga, one might postulate that [^18^F]FET-βAG-TOCA imaging could detect smaller lesions compared with [^68^Ga]Ga-DOTA-peptide imaging. On the 20 discordant lesions, 13 were detected only on [^18^F]FET-βAG-TOCA, and all were less than or equal to 10 mm in size, whereas 7 were detected only on[^68^Ga]Ga-DOTA-peptide imaging, of which all were greater than or equal to 8 mm in size except for 1 (Table 2). The latest digital PET detector technology may improve detection of small lesions.

The use of [^18^F]FET-βAG-TOCA PET/CT may be considered in the clinical setting where difficulties accessing [^68^Ga]Ga-DOTA-peptide have led to longer waiting times for patients, particularly where delivery of [^68^Ga]Ga-DOTA-peptide is limited to those centers within close proximity to the gallium generator. Delivery of low yields of [^68^Ga]Ga-DOTA-peptide is also a common problem and can lead to last minute cancellation of scanning slots with an ever-increasing burden on nuclear medicine departments. Recent work has explored the utility of [^18^F]F-AIF-1,4,7-triazacyclononane-1,4,7-tri-acetate-octreotide ([^18^F]F-AIF-NOTA-octreotide compared with [^68^Ga]Ga-DOTATATE/NOC in patients with NET (17). In this study the noninferiority of ^18^F-labeled AIF-NOTA-octreotide was illustrated; the authors reported high physiologic uptake in the pancreas, necessitating the need for additional cross-sectional imaging to delineate any pancreatic lesion, a feature not observed with [^18^F]FET-βAG-TOCA (8). Moreover, SUV_max_ was lower than [^68^Ga]Ga-DOTATATE and TBR within the bone was particularly lower, which may have implications in assessing disease response to therapy within the bone. [^64^Cu]Cu-DOTATATE has also recently been studied, with comparable results to [^68^Ga]Ga-DOTATOC, albeit with a higher radiation burden, which may not be acceptable to users, particularly as patients typically undergo multiple PET/CT studies during their disease journey (18).

However, there are some key limitations. As [^18^F]FET-βAG-TOCA imaging was performed after [^68^Ga]Ga-DOTA-peptide imaging in most patients, potential sequence effects cannot be excluded, but most were performed within 6 mo and no change in treatment occurred between both scans. Moreover, due to the variation in time interval between the 2 scans, changes in tumor composition or size and the possible change in SSTR density cannot be excluded (14). Although most patients underwent [^68^Ga]Ga-DOTATATE imaging, a number were imaged with other [^68^Ga]Ga-DOTA-peptide radioligands, the impact of which remains unclear. Finally, PET findings were not validated by a reference imaging standard such that sensitivity or specificity cannot be established.

## CONCLUSION

In this prospective head-to-head comparison of [^18^F]FET-βAG-TOCA PET/CT and [^68^Ga]Ga-DOTA-peptide PET/CT we have shown excellent tumoural uptake and noninferiority at both lesion and regional levels. [^18^F]FET-βAG-TOCA could potentially be used clinically as an alternate to [^68^Ga]Ga-DOTA-peptide. Further developments could lead to its use as a theranostic agent in locally advanced and metastatic NET.

## DISCLOSURE

This work was funded by Medical Research Council Developmental Clinical Studies grant MR/J007986/1. The authors acknowledge infrastructure support from the National Institute for Health Research Imperial Biomedical Research Centre and the Imperial College Experimental Cancer Medicine Centre. Rohini Sharma received grant funding from Incyte, AAA, Boston Scientific, and Terumo. Mairéad McNamara received research grant support from Servier, Ipsen, NuCana, and AstraZeneca, travel and accommodation support from AAA, Ipsen, and AstraZeneca, and speaker honoraria from AAA and AstraZeneca and is on the advisory boards for Incyte and AstraZeneca. Andrea Frilling received grants from Novartis, AAA, Ipsen, Sirtex, and Clifton, all unrelated to the submitted work. Angela Lamarca received travel and educational support from Ipsen, Pfizer, Bayer, AAA, SirtEx, Novartis, Mylan, Delcath Advanz Pharma, and Roche, speaker honoraria from Merck, Pfizer, Ipsen, Incyte, AAA/Novartis, QED, Servier, AstraZeneca, EISAI, Roche, Advanz Pharma, and MSD, advisory and consultancy honoraria from EISAI, Nutricia, Ipsen, QED, Roche, Servier, Boston Scientific, Albireo Pharma, AstraZeneca, Boehringer Ingelheim, GENFIT, TransThera Biosciences, Taiho, and MSD, and principal investigator–associated institutional funding from QED, Merck, Boehringer Ingelheim, Servier, AstraZeneca, GenFit, Albireo Pharma, Taiho, TransThera, and Roche and is a member of the Knowledge Network and NETConnect Initiatives funded by Ipsen. Juan Valle received personal fees from Agios, AstraZeneca, Baxter, Genoscience Pharma, Hutchison Medipharma, Imaging Equipment Ltd (AAA), Incyte, Ipsen, QED, Servier, Sirtex, and Zymeworks and grants, personal fees, and nonfinancial support from NuCana, all outside the submitted work. Richard Hubner received consultancy fees from Beigene, Ipsen, and Novartis and travel and accommodation support from Roche. The views expressed are those of the authors and not necessarily those of the NIHR or the Department of Health and Social Care. No other potential conflict of interest relevant to this article was reported.

## Notes

KEY POINTS **QUESTION:** How does the novel fluorine-labeled PET tracer [^18^F]FET-βAG-TOCA compare with [^68^Ga]Ga-DOTA-peptide PET/CT for the detection of NET? **PERTINENT FINDINGS:** In a prospective, noninferiority study in 45 patients with histologically confirmed NET, we observed excellent correlations between both tracers with no difference across median SUV_max_. **IMPLICATIONS FOR PATIENT CARE:** [^18^F]FET-βAG-TOCA may be considered in routine clinical practice for imaging NET.
